# Supplementary material for: Treatment Response in Individual Organs Affected by Chronic Graft-Versus-Host Disease
Source: Cells. 2025 Feb 7;14(4):238. doi: 10.3390/cells14040238 (PMC11854124; doi:10.3390/cells14040238)
Supplement: Supplementary file 1 [file cells-14-00238-s001.zip › cells-3382926-supplementary.pdf]

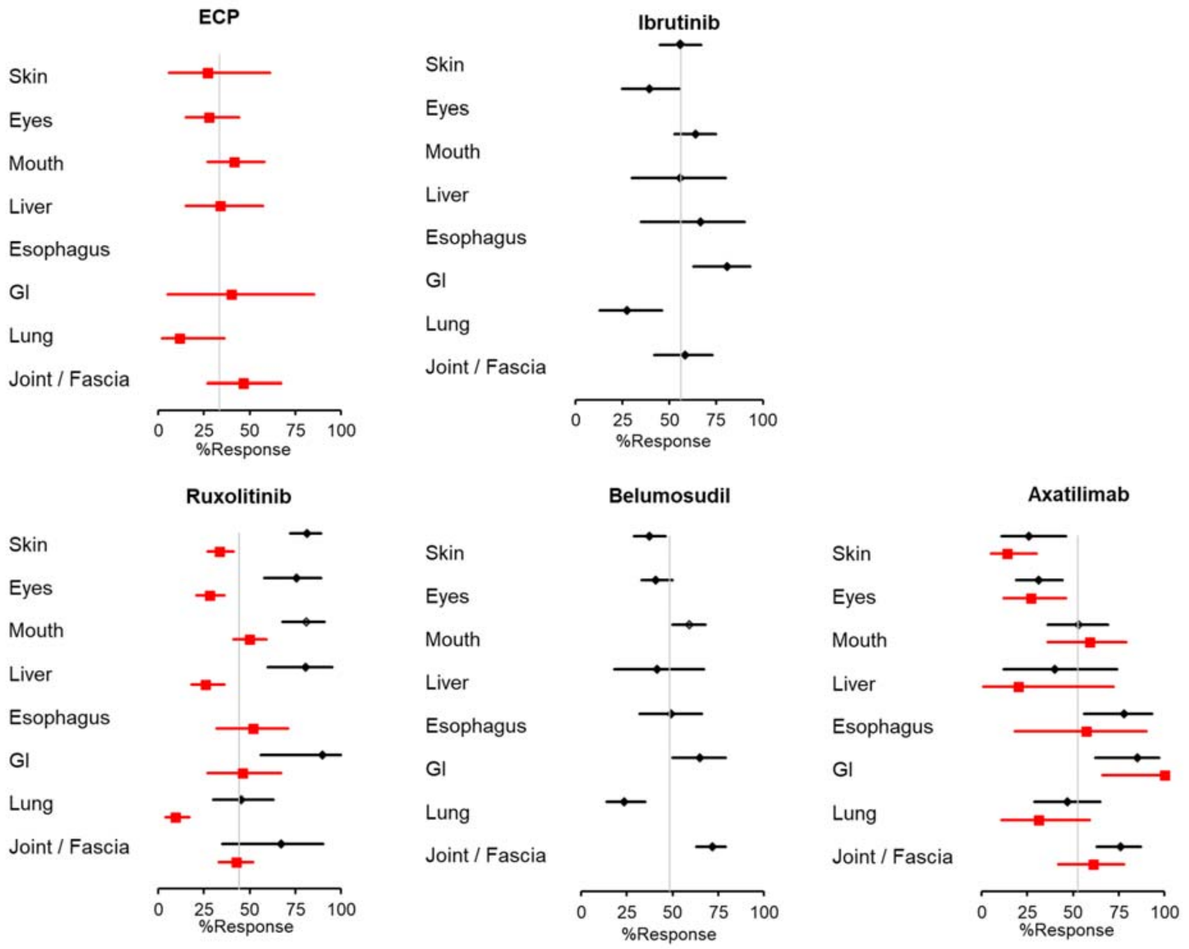

Figure S1. Pooled response rates at 24 weeks for individual organs according to drugs. Best response rates are shown in black and response rates at a fixed point of 24 weeks are shown in red. Diamonds and squares represent mean values with bars showing 95% confidence intervals. The vertical dotted line indicates overall means of all drugs regardless of which endpoint was used. ECP = extracorporeal photopheresis, GI = gastrointestinal. that met each criterion. ECP = extracorporeal photopheresis.
